# Supplementary figures and images for: Perineuronal Nets on CA2 Pyramidal Cells and Parvalbumin-Expressing Cells Differentially Regulate Hippocampal-Dependent Memory
Source: J Neurosci. 2024 Dec 31;45(6):e1626242024. doi: 10.1523/JNEUROSCI.1626-24.2024 (PMC11800750; doi:10.1523/JNEUROSCI.1626-24.2024)

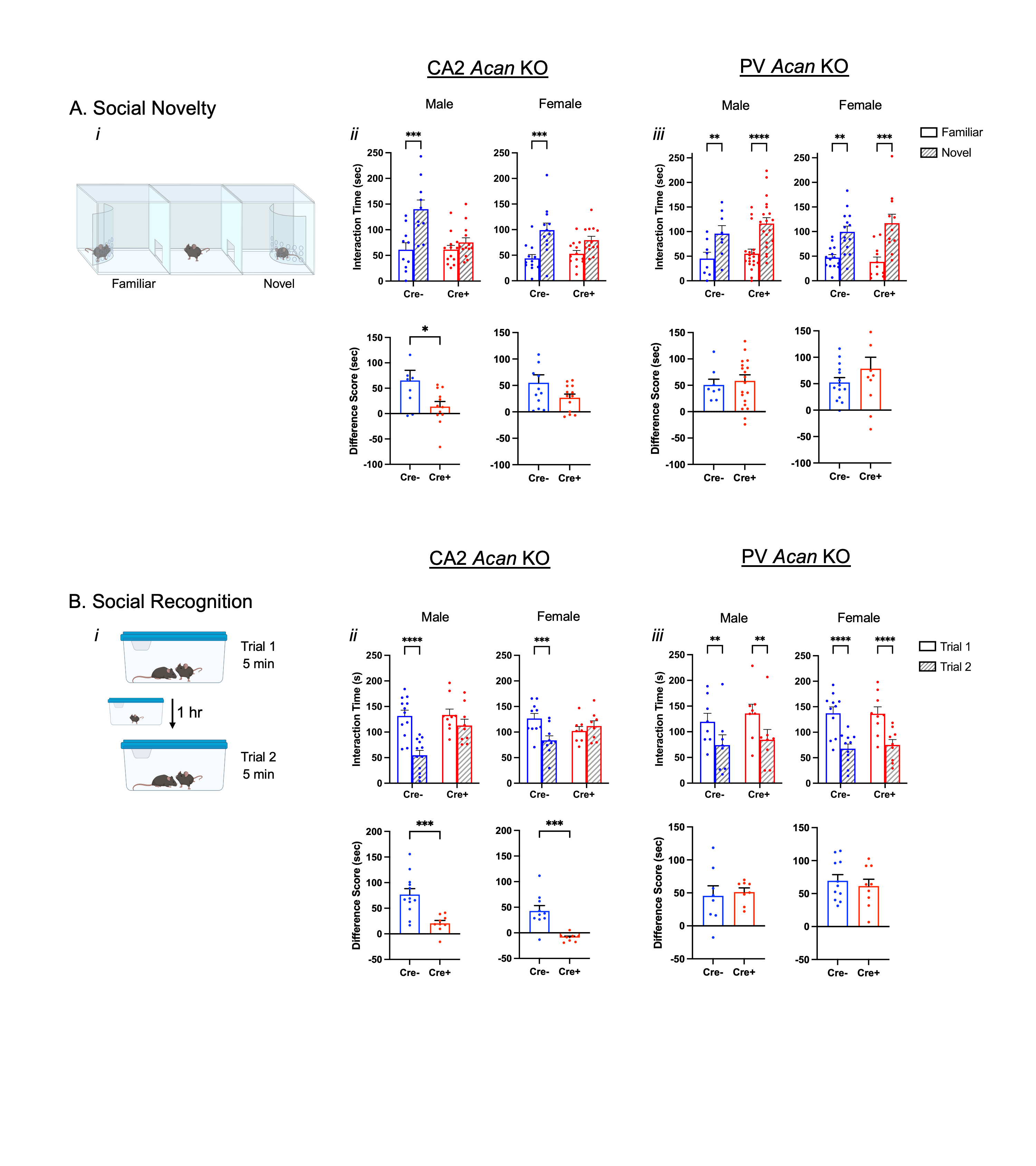

Supplement: Figure 2-1 — Amigo2 Acan KOs, but not PV Acan KOs, have impaired social recognition memory. A. In the preference for social novelty assay (i), animals have a choice between a novel and familiar mouse. Among CA2 Acan KO animals (ii), for both males and females, Cre- animals preferred the novel mouse but Cre + animals did not (males: main effect of chamber: F(1,21) = 17.6, p = 0.0004, main effect of genotype: F(1,21) = 6.3, p = 0.02, interaction: F(1,21) = 8.5, p = 0.008; post hoc: Cre-: p = 0.0002, Cre+: p = 0.60; females: main effect of chamber: F(1,23) = 26.01, p < 0.0001, main effect of genotype: F(1,23) = 0.28, p = 0.60, interaction: F(1,23) = 3.16, p = 0.088; multiple comparisons: Cre-: p = 0.0002, Cre+: p = 0.050). Difference scores (novel minus familiar) showed that Cre- males preferred the novel mouse significantly more than Cre + males, whereas difference scores were not significantly different for females (males: t(19) = 2.49, p = 0.022; females: t(23) = 1.78, p = 0.089). However, both Cre- controls and Cre + PV Acan KO animals preferred the novel mouse. Preference for the novel was seen in both males and females (males: main effect of chamber: F(1,25) = 33.69, p < 0.0001, main effect of genotype: F(1,25) = 0.89, p = 0.35; females: main effect of chamber: F(1,23) = 36.25, p < 0.0001, main effect of genotype: F(1,23) = 0.12, p = 0.72). Difference scores were similar between the two genotypes for both males and females (males: t(26) = 0.40, p = 0.69; females: t(23) = 1.21, p = 0.24). B. In the direct interaction test of social recognition memory assay (i), animals were exposed to a novel animal in trial 1 and the same, now-familiar, animal in trial 2. For the CA2 Acan KO strain (ii), for both males and females, Cre- controls spent less time with the stimulus animal on trial 2 than trial 1, but Cre + KOs spent equivalent time with the stimulus mouse in each trial (males: main effect of trial: F(1,19) = 46.75, p < 0.0001, main effect of genotype: F(1,19) = 4.51, p = 0.047; [file jneuro-45-e1626242024-s001.tif]

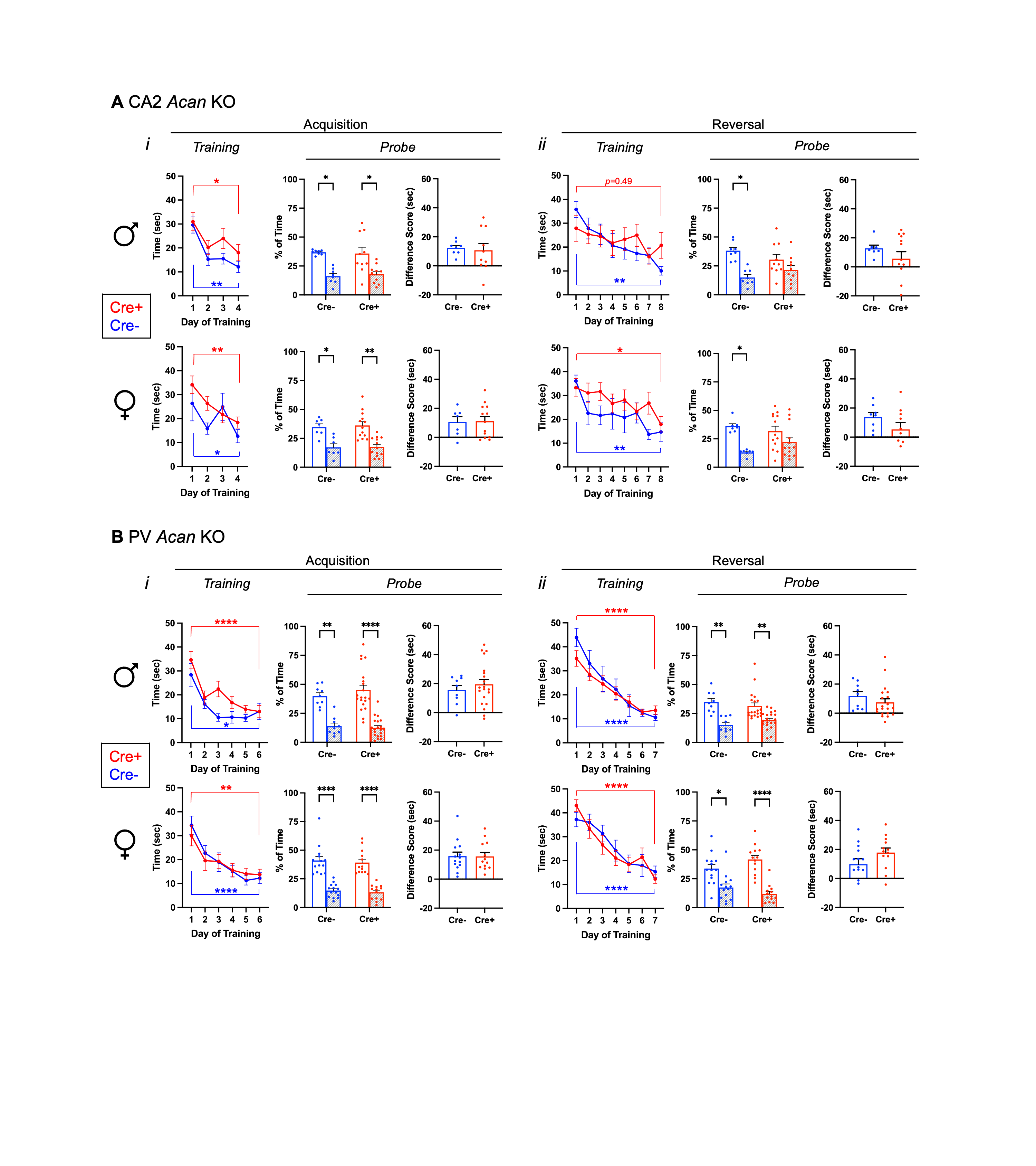

Supplement: Figure 4-1 — Morris water maze data for Amigo2 Acan KO (A) and PV Acan KO (B) animals. For each strain, data are split into that from males (top row) and females (bottom row) for the acquisition and reversal phases of the assay. For each phase, training data show the latency to reach the hidden platform over each day of training, and probe data show the percent of time that animals spent swimming in the quadrant of the pool where the platform was located during training and the opposite quadrant. Difference scores represent the time in the target quadrant minus time in the opposite quadrant. Ai, For Amigo2 Acan KO animals, both males and females showed no difference between Cre- and Cre + animals in latency to reach the platform during acquisition training (males: main effect of training day: F(3,48) = 8.51, p = 0.0001, main effect of genotype: F(1,16) = 4.16, p = 0.058; females: training day: F(3,54) = 6.2, p = 0.0011, genotype: F(1,18) = 2.02, p = 0.17), and all groups showed a significant reduction in latency to reach the platform from the first to the last day of training (males: Cre-: p = 0.0044, Cre+: p = 0.017; females: Cre-: p = 0.033, Cre+: p = 0.0039). Both Cre- and Cre + animals also spent significantly more time in the target quadrant than the opposite quadrant during the acquisition probe trial (males: quadrant: F(1,16) = 17.80, p = 0.0007, genotype: F(1,16) = 0.020, p = 0.89; multiple comparisons: Cre- p = 0.016, Cre + p = 0.019; females: quadrant: F(1,18) = 18.24, p = 0.0005, genotype: F(1,18) = 0.6179, p = 0.44; multiple comparisons: Cre- p = 0.038, Cre + p = 0.0033). Difference scores were similar between genotypes within each sex (males: t(11.2) = 0.33, p = 0.75; females: t(18) = 0.11, p = 0.92). Aii. In the reversal phase of the assay, for both males and females, a main effect of training day was detected (males: training day: F(7,112) = 4.91, p < 0.0001; genotype: F(1,16) = 0.11, p = 0.74; females: training day: F(7,126) = 3.96, p = 0.0006; genotype: F(1,18) [file jneuro-45-e1626242024-s002.tif]
